# 3D Printing Technique Assisted Autologous Costal Cartilage Augmentation Rhinoplasty for Patients with Radix Augmentation Needs and Nasal Deformity after Cleft Lip Repair

**DOI:** 10.3390/jcm11247439

**Published:** 2022-12-15

**Authors:** Haidong Li, Jingyi Wang, Tao Song

**Affiliations:** Plastic Surgery Hospital, Chinese Academy of Medical Sciences and Peking Union Medical College, Beijing 100144, China

**Keywords:** 3D printing, augmentation rhinoplasty, cleft lip nasal deformity, costal cartilage, transplantation

## Abstract

Objective: to better reconstruct the nasal shape after cleft lip repair with 3D printing assisted autologous costal cartilage augmentation rhinoplasty, especially for patients with radix augmentation needs. Method: 20 patients with nasal deformity secondary to cleft lip repair and radix augmentation needs had received surgical treatment from July 2016 to November 2021. A total of 10 cases were treated with autologous costal cartilage augmentation rhinoplasty for nasal deformity after cleft lip repair, and 10 cases were treated with the help of 3D printing. According to the characteristics of nasal deformity, autologous costal cartilage was carved and implanted into the nose back. Results: 3D printing assisted autologous costal cartilage augmentation in the treatment of nasal deformity after cleft lip repair, the incision healed well, and there were no complications in the thoracic cartilage donor area. The shape of the nose is satisfactory, the height and shape of the nose tip and the size of both nostrils are mostly symmetrical, the nasal columella is elongated, the original nose tip is flat, the collapse of the nose wing is satisfactory, and the nose lip angle is close to normal. Conclusions: 3D printing assisted autologous costal cartilage augmentation is an ideal treatment for nasal deformity after cleft lip repair.

## 1. Introduction

Cleft lip is one of the most common congenital malformations in the maxillofacial region. The repair of cleft lip and nose deformity has important clinical significance in plastic and cosmetic surgery. Cleft lip deformity is not only the full-thickness dehiscence of the upper lip, but also accompanied by a series of nasal deformities, including the collapse of the nasal dorsum, low and flat nasal root, insufficient nasal length, blunt nasal tip, and the retraction of the nasal columella. The nasal deformity can have a strong effect on a patient’s appearance and mental health state. Severe nasal deformity even hinders the patients’ respiratory and pronunciation function. Nasal plastic surgeries after cleft lip surgery often choose rib nose comprehensive plastic surgery to obtain a better nasal shape [1].

Among them, autologous costal cartilage has the advantages of sufficient material and strong support. It can make various fine adjustments to the nasal tip support according to the needs and can effectively resist the contraction of the skin and obtain a more stable long-term effect. Moreover, since the nasal cartilage of East Asians is generally weak and the nasal septal cartilage is short, the nose tip shaping of autologous costal cartilage came into being and became popular among Asians. Especially in the case of secondary nasal surgery for secondary deformity of cleft lip, the scar contracture of alar cartilage makes autologous costal cartilage almost the only choice.

However, when autologous costal cartilage is used for dorsal nasal transplantation, the connection between costal cartilage and nasal bone is difficult to master. It is necessary to adjust the carved costal cartilage repeatedly during the operation. Otherwise, the costal cartilage will not be well connected with the nasal bone. The nasal bone of cleft lip deformity also has deflection deformity, which makes the connection part more difficult [2,3,4,5].

From July 2016 to November 2021, we used 3D printing assisted autologous costal cartilage rhinoplasty to treat nasal deformity after cleft lip repair. After cleft lip repair, the nasal back does not get deformed and the operation time has no significant increase. The nasal deformity has achieved good results and met the patients’ demands. The report is as follows.

## 2. Clinical Data

### 2.1. Patients

A total of 20 patients with nasal deformity after congenital cleft lip repair surgery were treated including 5 males and 15 females. The age ranged from 18 to 27 years, with an average of 20.6. There were 11 cases of left cleft lip, 7 right cleft lip, and 2 bilateral cleft lip. The main deformities including low and flat nasion, insufficient nasal length, blunt nasal tip and retraction of the nasal columella. All the patients were unsatisfied with the low nose bridge and wanted to do the radix augmentation operation at the same time.

The patients were randomly divided into 2 groups. One group used ordinary costal cartilage rhinoplasty, and the other used 3D printing assisted autologous costal cartilage rhinoplasty for the treatment (Table 1).

### 2.2. Surgical Procedures

The conventional treatment group included the following procedures: After general intravenous anesthesia, the patient took the supine position. A 5 cm incision was made on the right costal margin to cut the 7th or 8th costal cartilage segment. We carved the cartilage according to the needed shape preparing for the nasal tip scaffold and nasal tip cartilage complex.

A bird-shaped incision was designed from the nasal columella and its base, extending to the outer alar foot of the affected side. The flap was lifted and separated to expose the alar cartilage completely. Suture the graft with the medial feet of the bilateral alar cartilage and the superior lateral cartilage of the septal cartilage. The costal cartilage was carved into an ideal shape and placed into the subfascia space of the nasal dorsum. The lower lateral cartilages and nasal valves were addressed. The septoplasty and the caudal extension grafting were performed. The nasal columella and the prosthetic head side and medial foot are raised with the nasal tip. After adjusting the coverage of cartilage in its natural state, the wound is sutured.

Both nostrils were fixed outside with a thermoplastic splint. Filled in the nostrils with Vaseline gauze, a drainage tube, and packing gauze inside for 24 h. Antibiotics, hemostasis, detumescence, and other drugs were used for 7 days till the suture and external fixation were removed.

**Figure 1:**
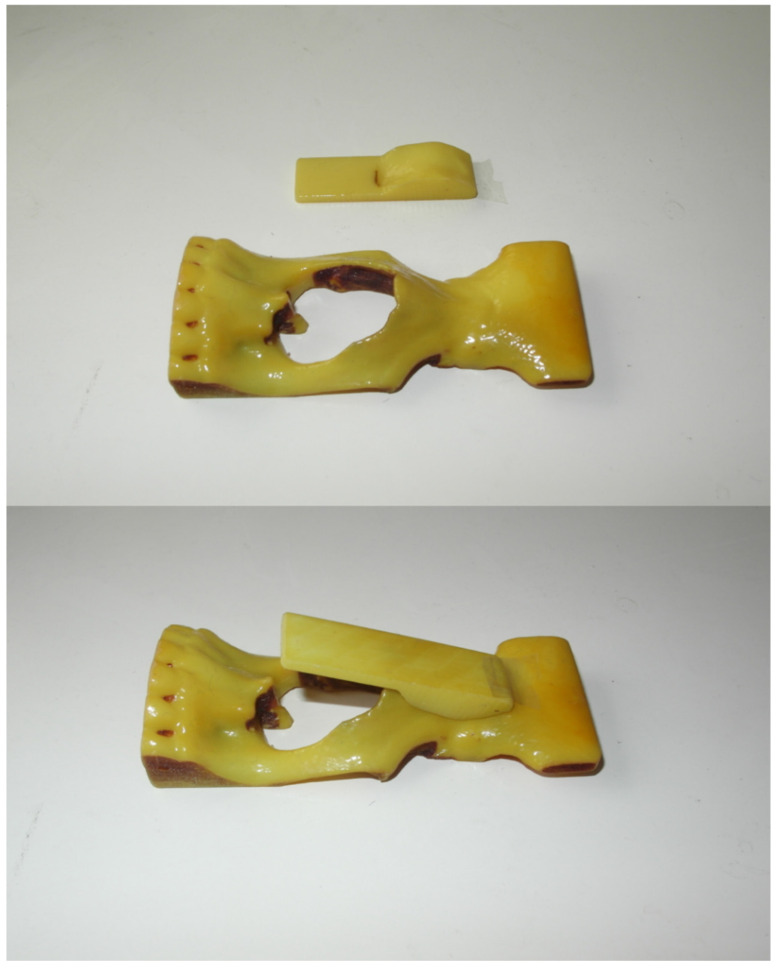
An example of the 3D printing model of PLA material. We can use the model to help to simulate the surgical scene.

**Figure 2:**
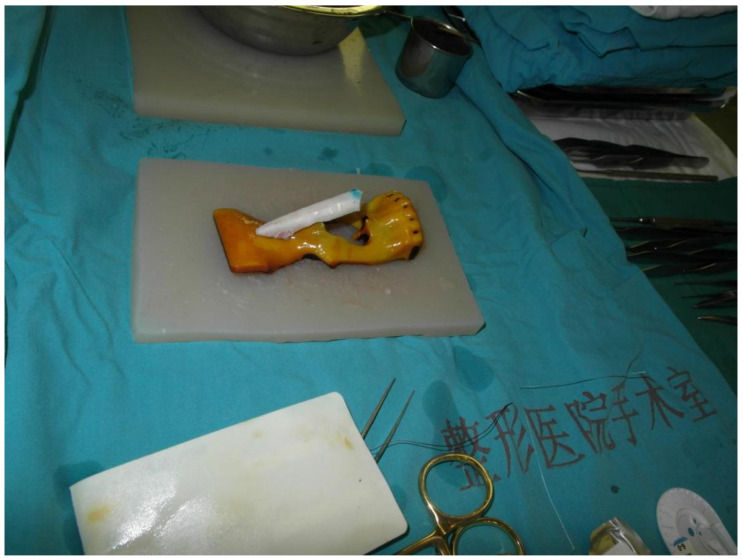
After the 3D printing model was sterilized, we put the carved ribs along with it during the operation.

When it comes to the 3D printing assisted autologous costal cartilage rhinoplasty group, patients took CT scans of the head and chest before the operation at first. Printed out the CT results in 3D (objet 30, STRATASYS, Israel), and printed the light cured resin material and simple PLA material, respectively (Figure 1). Before the operation, 3D software was used to make an impression for comparison during the surgery. Carrots were used to simulate the costal cartilage to carve before surgery. After engraving, it is placed on the 3D printing model of PLA material. Using the carrot prosthesis and printing model, we can perfectly simulate the surgical scene to make nasal root radian anastomosis the prosthesis. We used sterilized light cured 3D printing model during the operation and simulated the carved ribs with it (Figure 2). Other surgical methods were the same as the control group.

## 3. Results

All patients were followed up for 6–22 months with an average of 9 months. After augmentation rhinoplasty with autologous costal cartilage assisted by 3D printing, all incisions were primary healing and no complications in the donor cartilage happened. Patients were satisfied with the appearance. 

**Figure 3:**
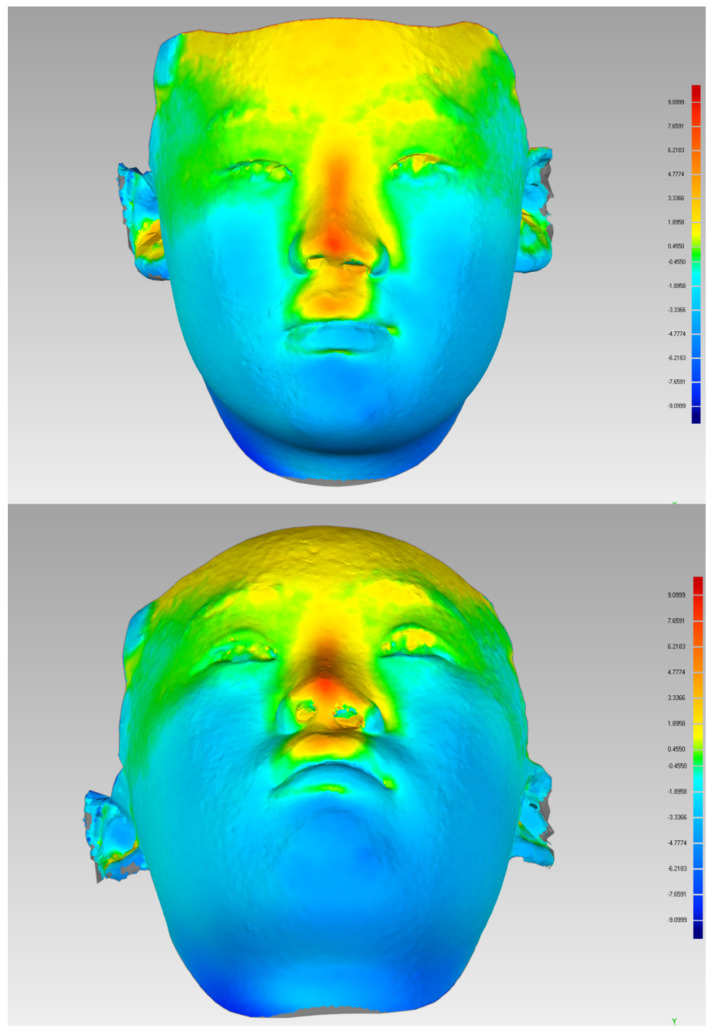
Three dimensional photographs were taken by three-dimensional photogrammetric camera (3dMDFaceTM System, 3dMD, USA) with a standardized protocol. Import images into Geomagic wrap software (Geomagic Wrap 2015, 3D Systems, USA) for thermographic analysis. Comparing the images before and after the operation after alignment registration, the patient’s nasal tip and nasal back changed greatly compared with that before the operation.

By using a three-dimensional photogrammetric camera (3dMDFaceTM System, 3dMD, Atlanta, GA, USA), 3D photographs were taken to compare the images before and after the operation (Figure 3). The height of the nasal tip and the shape of both nostrils are basically symmetrical. The nasal columella was elongated, the nasal tip was flat, and the collapse of the nasal wing was recovered satisfactorily. A satisfactory nasal dorsal curve was obtained after the operation. The tip defining points and the supratip break was clear. There was no perforation of the nasal septum, no cartilage exposure, and no nasal ventilation, and the nasolabial angle was close to normal.

The average operation time was 179 min in patients with rhinoplasty assisted by 3D printing, while the control group took 247 min (Table 2). There was 1 patient with costal cartilage augmentation rhinoplasty that got infected, recovered after drainage and antibiotic treatment, and 2 patients received secondary operation treatment because of the warpage of costal cartilage after the treatment. No patients in the 3D printing group got infected or found the cartilage improper, and no complications such as warping and contour irregularity were reported.

## 4. Case Report

**Figure 4:**
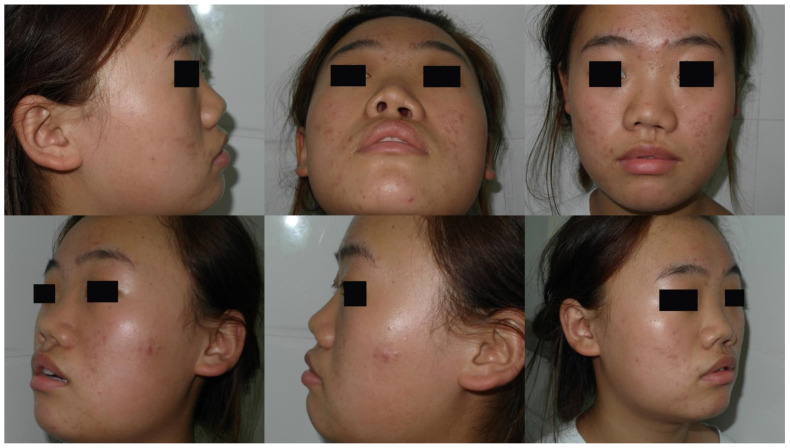
A 19-year-old female presented with cleft lip nasal deformity before our surgery. The photos were taken before the operation.

**Figure 5:**
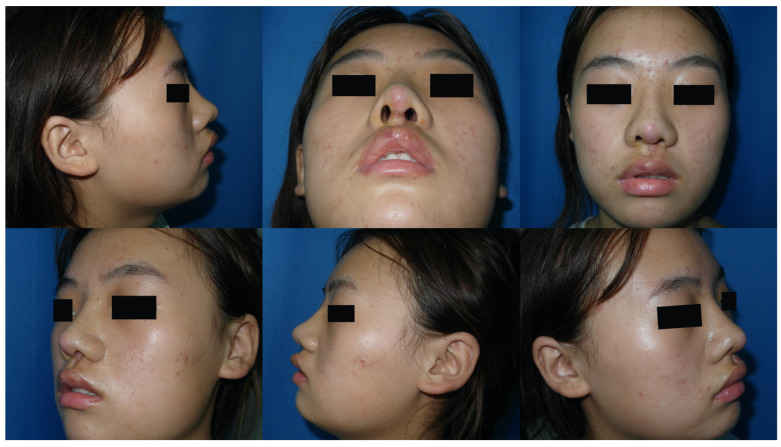
A 19-year-old female presented with cleft lip nasal deformity before our surgery. The photos were taken 6 months after the operation. Because of the premature absorption of the absorbable line, the nostrils were not exactly symmetrical. Further surgery could be taken to tighten the muscles of the nasal basis on the surgical side later in order to make the appearance more symmetrical.

Case 1: A 19-year-old female presented with cleft lip nasal deformity before our surgery. She received the 3D printing technique assisted autologous costal cartilage augmentation rhinoplasty. Figure 4 show the preoperative and 6 months postoperative images Figure 5. Because of the premature absorption of the absorbable line, the nostrils were not exactly symmetrical. Further surgery could be taken to tighten the muscles of the nasal basis on the surgical side later in order to make the appearance more symmetrical.

**Figure 6:**
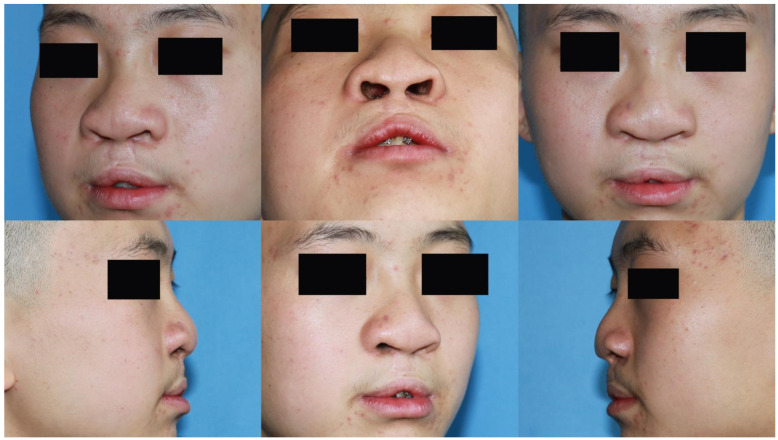
A 25-year-old male presented with cleft lip nasal deformity before our surgery. The photos were taken before the operation.

**Figure 7:**
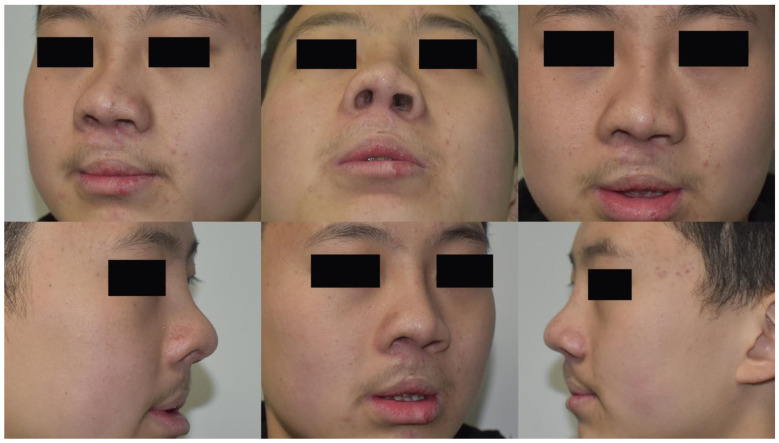
A 25-year-old male presented with cleft lip nasal deformity before our surgery. The photos were taken 12 months after the operation.

Case 2: A 25-year-old male presented with cleft lip nasal deformity before our surgery. He received the 3D printing technique assisted autologous costal cartilage augmentation rhinoplasty. Figure 6 show the preoperative and 12 months postoperative images are shown in Figure 7.

## 5. Discussion

Nasal deformity after cleft lip repair is currently regarded as the result of both tissue displacement and hypoplasia. Patients in our study were found not only to have the distortion of alar cartilage, but also the collapse and deflection deformity. Therefore, the perfect fitting of graft material to nasal bone is the key to the success of the operation.

In traditional surgery, autologous costal cartilage is used for dorsal nasal transplantation and it is difficult to well connect the costal cartilage and nasal bone, especially for patients with nasal bone deflection deformity. The surgeon often needs to repeat adjusting the shape of the cartilage during the surgery. The repeated adjustment elongates the incision exposure time, and accordingly increases the probability of infection [6]. In some cases, to finish the operation in a shorter time, the protruding nasal bone needs to be shaped till the upper platform becomes flat, which will also increase the pain of patients and the blood loss. Moreover, if the patient is unsatisfied with the surgery effect and wants to return to its preoperative stage, the nose will be lower and flatter than before [7].

Autologous costal cartilage used in nasal dorsum transplantation owns the advantages of stable biological properties, good histocompatibility, stable postoperative appearance and natural radian. For patients with nasolabial deformity secondary to cleft lip surgery, using autologous costal cartilage for the surgery has added advantages including cartilage being easier to survive, more convenient material selection, less rejection and deformation, and better effect. It can also avoid the pain of taking multiple operations and reduce the burden of economy. Autologous costal cartilage transplantation with fewer postoperative complications and a lower cost has been widely adopted nowadays. However, both autologous costal cartilage and nasal bone are rigid materials and cannot be deformed easily such as silicone materials. If the cartilage was trimmed improperly on the nasal back, rib deflection and seesaw-like deformity will be formed, and it may cause poor bone healing and requires secondary surgery for adjustment [8].

Using the 3D printing technique to assist autologous costal cartilage augmentation rhinoplasty owns many advantages. First of all, the core of the nasal alar stent is costal cartilage with good histocompatibility. It can offer strong support to fully correct the collapse deformity. Moreover, 3D printing helps to better utilize costal cartilage, making the size of carved costal cartilage more appropriate for every patient. The stress of cartilage and the skin tension of the nasal tip are reduced. Correspondingly, this reduces the risk of the movement or shaking of the graft and deformity or skin collapse after the operation. In addition, the use of 3D printing reduced the time spent on repeated adjustment for the cartilage shape as much as possible, which can greatly reduce the operation time as well as the risk of infection. Lastly, we used carrots to simulate operation because the hardness of carrots is close to the costal cartilage. When the carved carrots are placed on the 3D printed nasal bone model, it can stage the operation scene well, making the costal cartilage better fit the nasal bone and avoid the seesaw-like deformity happens.

To sum up, cleft lip patients generally have serious nasal deformities accompanied by the distortion of nasal septum and nasal bone. If 3D printing can be used to assist autologous costal cartilage augmentation rhinoplasty, the deflection of costal cartilage can well correct and achieve satisfied results.

## Figures and Tables

**Table 1:** The baseline information and the follow up time of the patients were recorded below.

	3D Printing Assistant (N = 10)	Simple Costal Cartilage (N = 10)	Overall (N = 20)
factor (sex)			
Female	7 (70%)	8 (80%)	15 (75%)
Male	3 (30%)	8 (20%)	5 (25%)
age (years)			
Mean (SD)	20.9 (2.85)	20.3 (2.79)	20.6 (2.76)
Median [Min, Max]	20.5 [18.0, 27.0]	20.0 [18.0, 27.0]	20.0 [18.0, 27.0]
factor (side)			
Bilateral	1 (10.0%)	1 (10.0%)	2 (10.0%)
Left	6 (60.0%)	5 (50.0%)	11 (55.0%)
Right	3 (30.0%)	4 (40.0%)	7 (35.0%)
Follow Up (months)			
Mean (SD)	9.30 (5.25)	8.70 (3.71)	9.00 (4.44)
Median [Min, Max]	6.00 [6.00, 22.0]	6.00 [6.00, 16.0]	6.00 [6.00, 22.0]

**Table 2:** The operation time of different groups were recorded below.

	3D Printing Assistant (N = 10)	Simple Costal Cartilage (N = 10)	Overall (N = 20)
Operation time (minutes)			
Mean (SD)	179 (33.1)	247 (46.0)	213 (52.3)
Median [Min, Max]	185 [120, 220]	245 [180, 320]	200 [120, 320]

## Data Availability

Not applicable.
